# Individual and combined associations of physical activity and cognitive function with all-cause mortality in older men and women: a prospective analysis of the German National Cohort (NAKO)

**DOI:** 10.1186/s12877-026-07357-2

**Published:** 2026-03-26

**Authors:** Daniela Georges, Elena Rakuša, Heiko Becher, Berit Brandes, Hermann Brenner, Volker Harth, Antje Hebestreit, Jana-Kristin Heise, Florian Herbolsheimer, Thomas Keil, Jasmin Kiekert, Lilian Krist, Michael Leitzmann, Claas Lendt, Rafael Mikolajczyk, Ute Mons, Ulrich Mueller, Nadia Obi, Annette Peters, Regina Pickford, Tobias Pischon, Börge Schmidt, Karen Steindorf, Andrea Werdecker, Gabriele Doblhammer

**Affiliations:** 1 Faculty of Economic and Social Sciences, University of Rostock, Rostock, Germany; 2 Rostock University Medical Centre, Rostock, Germany; 3 German Center for Neurodegenerative Diseases, Bonn, Germany; 4 Heidelberg Institute of Global Health, Heidelberg, Germany; 5 University Hospital Heidelberg, Heidelberg, Germany; 6 Leibniz Institute for Prevention Research and Epidemiology - BIPS, Bremen, Germany; 7 Cancer Prevention Graduate School, German Cancer Research Center (DKFZ), Heidelberg, Germany; 8 Institute for Occupational and Maritime Medicine (ZfAM), University Medical Center Hamburg-Eppendorf, Hamburg, Germany; 9 Department of Epidemiology, Helmholtz Centre for Infection Research (HZI), Brunswick, Germany; 10 Division of Physical Activity, Cancer Prevention and Survivorship, German Cancer Research Center (DKFZ), Heidelberg, Germany; 11 Institute of Social Medicine, Epidemiology and Health Economics, Charité – Universitätsmedizin Berlin, Berlin, Germany; 12 Institute of Clinical Epidemiology and Biometry, University of Würzburg, Würzburg, Germany; 13 Institute for Prevention and Cancer Epidemiology, Faculty of Medicine, University of Freiburg, Freiburg, Germany; 14 Institute of Epidemiology and Preventive Medicine, University of Regensburg, Regensburg, Germany; 15 School of Sport and Recreation, Auckland University of Technology, Auckland, New Zealand; 16 Institute for Medical Epidemiology, Biometrics and Informatics, Medical Faculty of the Martin Luther-University Halle-Wittenberg, Halle (Saale), Germany; 17 Division of Primary Cancer Prevention, German Cancer Research Center (DKFZ), Heidelberg, Germany; 18 Institute for Health Services Research and Clinical Epidemiology, Philipps-University Marburg, Marburg, Germany; 19 Institute of Epidemiology, Helmholtz Zentrum München - German Research Center for Environmental Health (GmbH), Neuherberg, Germany; 20 Chair of Epidemiology, Institute for Medical Information Processing, Biometry and Epidemiology, Medical Faculty, Ludwig-Maximilians-Universität München, Munich, Germany; 21 German Center for Diabetes Research (DZD E.V.), Neuherberg, Germany; 22 German Centre for Cardiovascular Research (DZHK), Partner Site Munich Heart Alliance, Munich, Germany; 23 German Center for Mental Health (DZPG), Partner Site Munich, Munich, Germany; 24 Max Delbrück Center for Molecular Medicine in the Helmholtz Association (MDC), Molecular Epidemiology Research Group, Berlin, Germany; 25 Max Delbrück Center for Molecular Medicine in the Helmholtz Association (MDC), Biobank Technology Platform, Berlin, Germany; 26 Institute for Medical Informatics, Biometry and Epidemiology, University Hospital of Essen, University of Duisburg-Essen, Essen, Germany; 27 Federal Institute for Population Research, Mortality-Follow-Up of German National Cohort (GNC), Wiesbaden, Germany

**Keywords:** Cognitive function, Physical activity, Sex differences, Healthy aging

## Abstract

**Objectives:**

Low physical activity (PA) and poor cognitive function are associated with higher mortality risks. However, little is known about their interaction, including whether PA may moderate cognition-related mortality risks. This study examines the combined associations of PA and cognition with all-cause mortality, with attention to sex differences.

**Methods:**

Using data from the German National Cohort and its mortality follow-up, we analyzed mortality risk based on: a) baseline low vs. sufficient PA (assessed via the global physical activity questionnaire using a threshold of < vs. ≥ 600 MET-minutes/week), b) baseline low vs. medium vs. high semantic memory (SM) and executive function/processing speed (EF/PS), assessed through factor analyses of a neurocognitive test battery, and c) their interaction on mortality in individuals aged 65 + up to 10 years of follow-up (N = 28,892). Cox models were estimated both in the total sample and stratified by sex, adjusting for relevant confounders and reporting both distinct and combined associations.

**Results:**

During follow-up, 1,605 individuals (5.6%) died: 1,097 men (7.5%) and 508 women (3.6%). Compared to individuals with low cognitive function, those with high SM (Hazard Ratio (HR) = 0.83 [95%CI: 0.67–1.02]), as well as high EF/PS (HR = 0.66 [0.53–0.83]) and medium EF/PS (HR = 0.68 [0.60–0.78]) had lower mortality risks. PA was associated with a 29% decreased mortality risk (HR = 0.71 [0.62–0.82]) compared to low PA. PA moderated the elevated risk from low cognition, with regard to EF/PS (low EF/PS*PA: HR = 0.65 [0.50–0.84] vs. low EF/PS*low PA: HR = 1 (ref.)) and SM (low SM*PA: HR = 0.60 [0.46–0.77] vs. low SM*low PA: HR = 1 (ref.)). The associations did not differ between men and women.

**Conclusion:**

Maintaining cognitive function and PA in older age is relevant for reducing mortality risk in both men and women. PA may offset risks linked to low cognition in both sexes, though mechanisms require further study.

**Trial registration:**

Clinical trial number: not applicable.

**Supplementary Information:**

The online version contains supplementary material available at 10.1186/s12877-026-07357-2.

## Background

Mortality is a pivotal public health outcome influenced by myriad factors, including cognitive function, which affects long-term health [31]. Cognitive impairment has been recognized as a significant predictor of elevated mortality risk in numerous studies [2, 31, 34, 58]. This association is partly attributable to cognitive decline, impairing individuals’ ability to manage their health and adhere to treatments, which can exacerbate chronic conditions [66]. Furthermore, cognitive impairment might be an early predictor of dementia [27], and has been associated with an increased risk of frailty and a broader systemic health deterioration [74], as well as an elevated risk of social isolation, depression, and a cessation of health-promoting activities [27, 49].

Growing evidence indicates that the strength of the association between cognitive function and mortality varies by cognitive domain [27, 29, 57, 58]. Across multiple cohort studies assessing different cognitive domains, verbal fluency (semantic retrieval) and processing speed/executive function consistently stand out as the strongest predictors of health trajectories and mortality, even when compared with episodic memory or general cognitive measures [12, 20, 22]. Executive function, which governs planning and executing goal-directed behaviors, reasoning, and judgment, demonstrates the strongest association with mortality risk, likely due to its critical role in maintaining independence and managing complex health behaviors [29]. Memory function, involving the encoding, storage, and retrieval of information, predicts mortality with more variable and by tendency weaker impact [21, 27]. Memory likely affects mortality through early neurodegeneration and the capacity to recall medical instructions and health-related cues [27]. These domain-specific patterns elucidate that not all cognitive aspects possess equal prognostic significance.

As cognitive decline typically commences in the mid-60s [70, 73] and life expectancy continues to rise, cognitive impairment is becoming increasingly prevalent in aging populations. The etiology of (unhealthy) cognitive decline is multifactorial, shaped by health behaviors, and comorbidities across the lifespan [11, 55]. Consequently, cognitive function serves as an independent predictor of mortality and may interact with lifestyle factors.

In recent decades, considerable shifts in environmental conditions, occupational demands, and patterns of leisure activities have contributed to significant changes in physical activity (PA) behaviors [52, 62]. Physical inactivity has emerged as a critical health concern, often referred to as a global pandemic [33]. Around one-third of the global adult population does not engage in sufficient PA [24, 52, 62]. Low PA contributes to increased mortality through mechanisms like impaired cardiometabolic regulation, increased inflammation, sarcopenia, and reduced neuroplasticity [35, 54], and is a major risk factor for non-communicable diseases, including cardiovascular diseases, diabetes, and cancers [14, 15, 38]. Furthermore, low PA has been linked to cognitive decline, possibly due to reduced cerebral blood flow, lower brain-derived neurotrophic factor levels, and less exposure to cognitively stimulating environments [3, 17]. Conversely, poor cognitive function may lead to lower PA levels through executive dysfunction, reduced motivation, or depressive symptoms [19]. Consequently, low PA and low cognitive performance not only emerge as independent determinants of mortality, potentially exerting reciprocal influences and interactively increasing mortality risk. Individuals who exhibit both low PA and impaired cognitive function experience the greatest mortality risk [18, 41].

Sex differences play a crucial role in the interplay between cognitive abilities, PA, and mortality. Women have longer lifespans but experience a higher morbidity in later life [50]. Cognitive aging also differs by sex,women tend to possess greater cognitive reserve but may face faster post-menopausal cognitive decline [40]. The association between cognition and mortality partly differs by sex [63], as do the magnitude and effects of PA [10, 13, 61]. Despite these differences, the combined impact and underlying mechanisms linking cognitive function, PA, and mortality – especially by sex – remain insufficiently understood.

This study examines the relationship between executive and memory function and all-cause mortality, with a focus on whether these associations are modified by PA. The analysis places particular emphasis on the nature and strength of these relationships across cognitive domains and adopts a sex-sensitive perspective. The primary research questions guiding this study are as follows: (1) How are executive and memory function associated with mortality risk in older adults? (2) Does PA moderate the mortality risk associated with poor cognition? (3) Do these associations differ by sex? It is hypothesized that executive dysfunction is more strongly associated with increased mortality than memory impairment, and that PA has a particularly beneficial effect on individuals with low cognitive levels. Moreover, it is anticipated that these effects will vary according to sex.

## Methods

### Data source

This study used data from the initial examinations of the German National Cohort (NAKO) carried out between 2014 and 2019, and the longitudinal Mortality Follow-Up (MoFU) of the NAKO. The NAKO is a multidisciplinary, population-based prospective cohort study that investigates widespread diseases, their risks and protective factors as well as prevention measures in the general population aged 19 to 74 years in Germany [53]. The NAKO examined over 200,000 men and women in 18 study centers in Germany. Examinations included face-to-face-interviews, self-administered touchscreen questionnaires and biomedical examinations [53]. In addition to life circumstances and disease biographies, PA measurements and a brief neurocognitive test battery have been implemented in the NAKO [32, 39].

### Mortality

The NAKO MoFU collected and validated death certificates, cause(s) of death and dates of death via case-by-case tracking of deceased subjects. This process involved active and passive follow-up procedures, including a regular rolling vital status survey of all study participants via a health follow-up questionnaire and the utilization of secondary data sources, such as inquiries at the registry offices or health authorities, death certificates, or other pertinent documentation such as from cancer registries or health insurance companies [36]. All events (examination and death) were assigned to the middle of the month. The survival time was calculated as the interval between December 2023 or month and year of death and the month and year of the initial examination. For those who survived, the survival time ranged between 51.5 months to 117.5 months, and for those who died, it ranged from 0.5 to 112.5 months. Five deceased participants with missing information regarding the year of death were excluded from the analyses.

### Measures of cognitive function

Cognitive function was assessed using a brief neurocognitive test battery. Our approach represents a modified version of the procedure described by Kleineidam et al. [32]. The present analyses incorporated six tests: two immediate word list recall trials, which assess the ability to recall words from a digitally recorded and presented list of 12 words,one delayed 12-word list recall trial; an animal name test, which requires enumerating as many animal names as possible within one minute; and two Stroop color-word tasks. To ensure conceptual consistency across indicators, the two Stroop color-word task variables – originally scaled such that higher values reflected lower cognitive function – were reverse-coded accordingly. Thus, higher scores indicate superior cognitive function across all measures. To identify latent cognitive domains, a principal component factor analysis was performed on the six cognitive test variables within the age group of 65 years and older, basically following the approach described by Kleineidam et al. [32]. Two distinct factors emerged: the first comprised the results of the three word recall trials, which reflect semantic memory (SM) (based on varimax rotated factors with scoring coefficients of 0.37–0.41). The second comprised the results of the animal names test and the two Stroop tasks, which reflect executive function and processing speed (EF/PS) (scoring coefficients of 0.34–0.52). All six test variables underwent z-standardization and age-standardization for the 65 + age group in our sample. The composite scores were determined by summing the standardized values for each indicator, creating age-specific measures that are comparable within this older population. In order to acknowledge the non-linear relationship between cognitive function and mortality [27], to include cases with missing data in the cognitive tests, and to include disparate categories with sufficiently high case numbers [59], cognition level categories were built. After conducting a thorough analysis of the domain-specific means and standard deviations (sd), the added scores were subsequently categorized as follows: low cognitive function (score ≤ mean - 1 sd), medium (mean – 1 sd ≤ score ≥ mean + 1 sd), and high (score ≥ mean + 1 sd). A missing category, including all cases with incomplete or unavailable data on the cognitive tests, was added.

### Physical Activity (PA)

The five sub-domains of PA recorded in the Global Physical Activity Questionnaire (GPAQ) – comprising self-reported moderate and vigorous PA at work as well as during leisure time and recreation, and PA when travelling to and from places within one week – have been combined and processed by the NAKO in accordance with the World Health Organization guideline [39, 72]. PA is quantified in minutes and weighted by intensity (moderate to vigorous) to estimate the total energy expenditure, expressed in metabolic equivalent of task (MET) minutes. In accordance with WHO recommendations, individuals engaging in less than 600 MET-minutes of PA per week were classified as low PA, while those reaching or exceeding 600 MET-minutes were classified as PA [72]. Additionally, a missing category was incorporated to encompass cases with partial or complete missing information on the GPAQ.

### Model specification

Within our models, we adjusted for a set of covariates beyond the two central exposures, with the objective of reducing confounding and isolating the independent associations [65]. The inclusion of age and sex was driven by prevailing scientific consensus, which consistently identified these factors as predictors of all-cause mortality in elderly populations and moderate the nexus of cognitive function, PA, and all-cause mortality [23]. Socioeconomic factors were added given robust associations between lower socioeconomic status and elevated mortality, poorer health and health behaviours, and constrained access to healthcare services [67, 68]. The potential for measurement bias, regional differences, and clustering effects was mitigated by controlling for language proficiency and study site [56, 64]. Lifestyle factors (smoking, body mass index, alcohol consumption) were included as known behavioural predictors of mortality and as potential confounders for both physical activity and health outcomes [42, 67]. Finally, the Charlson Comorbidity Index (CCI) was employed to adjust for the individual comorbidity burden. The CCI is a validated, weighted summary measure of multimorbidity that was originally developed to predict one-year mortality risk in patients. However, it has been demonstrated to be a versatile and robust predictor of broad health outcomes, not limited to short-term outcomes or specific patient groups [8].

### Measurement of covariates

We adjusted for and stratified by self-administered sex (male/female). Furthermore, we adjusted for sociodemographic characteristics: age (both the metric and the square of the metric, each in deviation from the mean age of 71.2 years), educational level (the highest educational qualification, allocated according to the International Standard Classification of Education (ISCED) 1997, and categorized as follows: low (level 0–2; pre-school, primary school, secondary school), medium (3–4; secondary school and vocational training completed), high (5–6; university degree, doctorate), and missing/incomplete information), income (categorized into quartiles and a missing/incomplete information category), German language proficiency (derived from self-reported indication on mother tongue and interviewer’s assessment, and categorized as follows: German as the mother tongue or (very) good German abilities; moderate or (very) weak German abilities; missing/incomplete information), and place of examination. Finally, the following two variables indicating the individual health status were included. The categorical covariate concerning major health-related lifestyle risks indicates the number of three unhealthy behaviors: current smoking, risky alcohol consumption (AUDIT-C score ≥ 4 in men or ≥ 3 in women), and obesity (body mass index > 30 kg/m^2^). The summarized integration of these three indicators, as demonstrated to be effective in predicting mortality, cognition, and PA, was driven by statistical and substantial considerations, namely their high intercorrelation and their reinforcing effects [6, 44]. The variable ranges from 0 to 3 and includes one category with missing data and incomplete information. Additionally, an adjusted CCI was included, encompassing the following self-reported comorbid conditions: myocardial infarction (weight, if applicable: 1), cardiac insufficiency or heart failure (1), stroke (1), intermittent claudication or arterial occlusive disease (1), chronic bronchitis, COPD or asthma (1), liver cirrhosis (1), diabetes mellitus (1), limited kidney function or chronic renal insufficiency (2), leukemia (2), other tumor diseases (2), and HIV infection or AIDS disease (6). The CCI score ranged from 0 to 12, with a higher score indicating a greater illness burden. The score was categorized as follows: 0, 1–2, 3–4, 5 and above, missing/incomplete information. No cases with missing information were excluded due to the exposures or covariates.

### Sample selection

Participants in the NAKO were randomly selected if they were 20–69 years old and lived in the study area; individuals unable to provide informed consent or participate in the examinations were excluded. Considering the age-related process of cognitive degeneration from the mid-60s onwards [70, 73] and the transition to retirement, the respondents included in this study are adults aged 65 and older (*N* = 28,897). Data on mortality was collected from the baseline visit date (between 2014 and 2019) until December 31, 2023 (follow-up period mean (SD): 46.4 months (28.0)). After excluding five subjects with missing data regarding the year of death, the final analyses encompassed 28,892 individuals, with 216,427 person-years (py) of observation.

### Statistical analyses

We adjusted our models for covariates that affect differences in cognition, PA or mortality risk. Except for age, all covariates were considered time-constant and derived from the baseline examination. Missing values, if any, were included as an additional category in all variables.

For descriptive statistics, the proportions were calculated, and Kaplan–Meier curves were derived for PA and the two cognition domains. For bivariate analyses, chi-square tests were conducted to examine the association between the outcome of follow-up all-cause mortality and the exposures, as well as the associations between the exposures. Cox proportional hazard regression models were employed to evaluate the association of multiple risk factors with the risk of mortality, with the resulting hazard ratios (HR), 95% confidence intervals (95%CI), and p-values being reported. Model 1 was adjusted for all variables. In order to examine interaction effects between cognitive function PA, interaction terms with categorical variables were included in models 2a and 2b. Interaction effects were interpreted by computing combined HR for each level of cognitive function, comparing low PA and sufficient PA, with statistical significance assessed using Wald tests. For easier interpretation of the association between PA and mortality across cognitive performance levels, within each cognitive level, the HR for low PA was set to 1, and the corresponding HR for sufficient PA was derived from the model estimates. Specifically, the HRs were obtained by combining the main effect of PA with the respective interaction term for each cognitive level from the model, i.e. HR_cognition level, lowPA_ = 1 (ref.), and HR_cognition level, PA_ = exp(β_PA_ + β_interaction(cognition level*PA)_). The effects (HRs and 95% CIs) were calculating using Stata’s postestimation lincom command. All analyses were conducted both for the analytic sample and stratified by sex. To ascertain whether the association between PA and cognitive function varied by sex, we expanded the Cox model to encompass a three-way interaction term between one cognitive domain, PA, and sex. For each model, the proportional hazards assumption was checked and verified using Schoenfeld residuals with the estat phtest command in Stata.

Furthermore, sensitivity analyses were conducted. These include the interaction models with a reduced set of covariates (models S1a/b: excluding socio-economic covariates, models S2a/b: excluding health-related covariates), continuous measures of cognitive function and PA (MET time) (models S3a/b), excluding individuals with a history of stroke (models S4a/b) or diabetes (models S5a/b) and right censoring starting after December 31, 2021 (models S6a/b). All analyses were conducted using Stata version 19.

## Results

### Descriptives and bivariate associations

Additional file 1 presents the descriptive statistics for the analytic sample (*N* = 28,892), men (*N* = 14,665), and women (*N* = 14,227). Overall, 5.6% of the total sample (*N* = 1,605) died within the follow-up period, 7.5% of the men (*N* = 1,097), and 3.6% of the women (*N* = 508).

The mean age at baseline was 67.4 years, with an almost balanced sex ratio (50.8% male, 49.2% female; Additional file 1). As anticipated, the majority demonstrated medium levels of cognitive function (EF/PS: 68.6%; SM: 65.6%). The proportion of high cognitive levels was greater in SM (14.9%) than in EF/PS (11.6%). Low PA was evident in 10.9%. Both the cognitive domains and PA as well as most of the covariates (with the exception of German language proficiency) were correlated with mortality (Additional file 1). Moreover, a bivariate association was observed between PA and the domains of cognitive function (each *p* < 0.001). A negative correlation was identified between low PA and an elevated frequency of low levels of cognition.

### Kaplan–Meier estimation

**Fig. 1:**
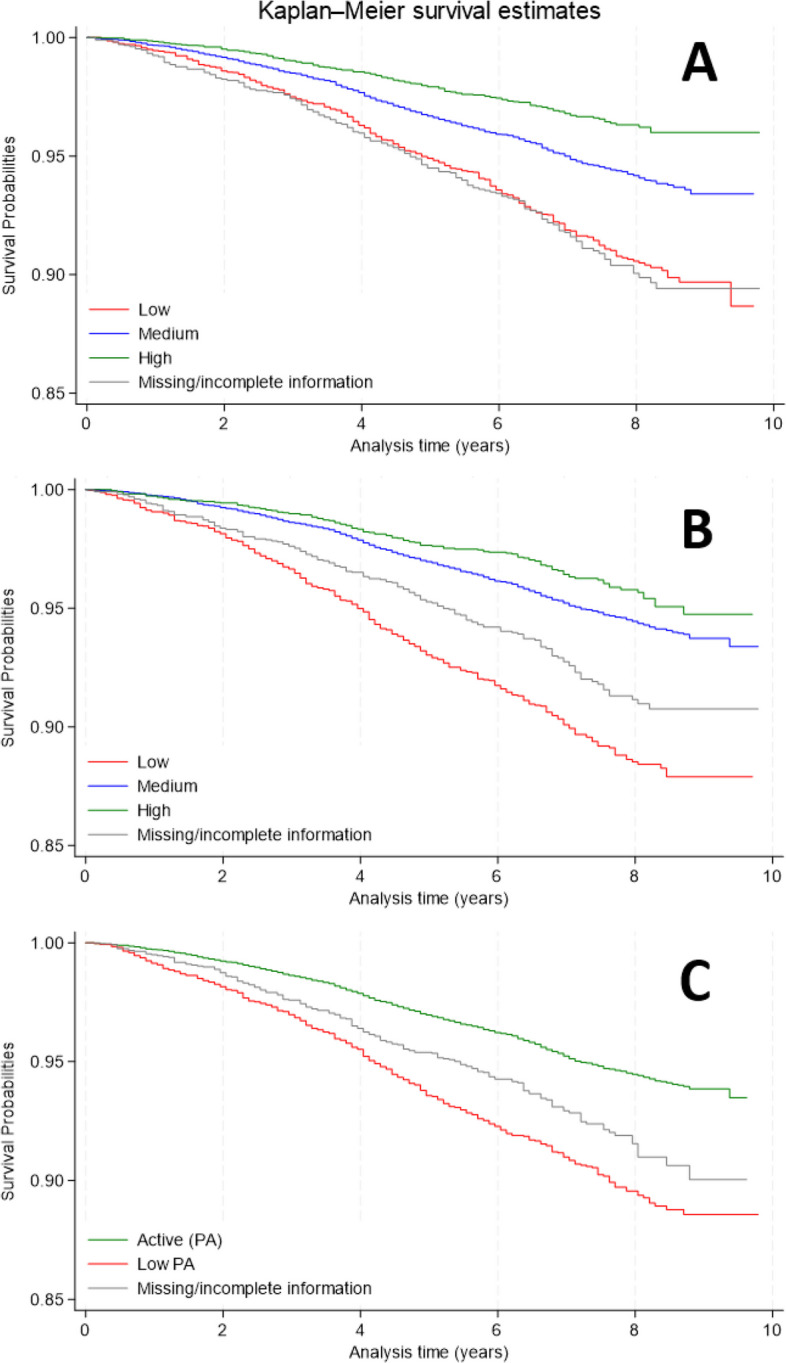
Kaplan–Meier plots of survival Note: **A** Semantic Memory (SM), **B** Executive Function/Processing Speed (EF/PS), **C** Physical activity (PA). Source: NAKO baseline and mortality follow-up (*N* = 28,892; py = 216,427) (own calculations)

In Fig. 1 survival probabilities according to SM, EF/PS, and PA are depicted. The lowest survival probabilities were associated with low cognitive function. At the end of the observation period, that is, after a maximum of ten years, the survival probabilities were as follows: 88.7% (95%CI: [86.1; 90.8]) in individuals with low SM, 93.4% [92.9; 93.9] in individuals with medium SM, and 96.0% [95.2; 96.7] in individuals with high SM. For EF/PS the probabilities were 87.9% [86.5; 89.2], 93.4% [92.5; 94.2], and 94.8% [93.4; 95.8], respectively. The 10-year survival probability of physically active individuals was 93.5% [92.6; 94.3] compared to 88.6% [87.1; 89.9] observed in low PA individuals.

### Cox proportional hazard models for the association between cognition, PA and mortality

Table 1 presents the findings of Cox proportional hazard models that investigated the association between cognition, PA and mortality for the analytic sample.

**Table 1:** Results of Cox proportional hazard model – distinct effects of cognition and PA (HR, p-values, 95%CI)

**Variables**		**M1—Distinct effects**			
		HR	p	lower 95%CI	upper 95%CI
Semantic Memory (SM)	Low (ref.)	1			
	Medium	0.92	0.184	0.81	1.04
	High	0.83	0.078	0.67	1.02
	Missing/incomplete	1.18	0.177	0.93	1.49
Executive function/processing speed (EF/PS)	Low (ref.)	1			
	Medium	0.68	< 0.001	0.60	0.78
	High	0.66	< 0.001	0.53	0.83
	Missing/incomplete	0.74	0.010	0.58	0.93
Physical Activity (PA)	Active (PA)	0.71	< 0.001	0.62	0.82
	Low PA (ref.)	1			
	Missing/incomplete	1.19	0.037	1.01	1.40
Subjects		28,892			
Failures (Deaths)		1,605			
Observations (py)		216,427			
Time at risk		201,534			
LR chi^2^		1,126			

*HR* hazard ratio, *95%CI* 95% confidence interval, *py* person-years, model adjusted for age, age^2^, sex, education, income, Charlson Comorbidity Index score, lifestyle risk index, German language proficiency, and place of examination. Source: NAKO baseline and mortality follow-up (*N* = 28,892; py = 216,427) (own calculations)

In comparison with low levels of SM, both medium (HR = 0.92 [0.81; 1.04]) and high levels (HR = 0.83 [95%CI: 0.67; 1.02]) of SM by tendency exhibited a slight decrease in mortality risk. Both medium (HR = 0.68 [0.60; 0.78]) and high levels (HR = 0.66 [053; 0.83]) of EF/PS were associated with a lower mortality risk compared to the low group (HR = 1). PA demonstrated a 29% decreased mortality risk (HR = 0.71 [0.62; 0.82]) compared to low PA (HR = 1). The results of the full model are presented in Additional file 2.

**Fig. 2:**
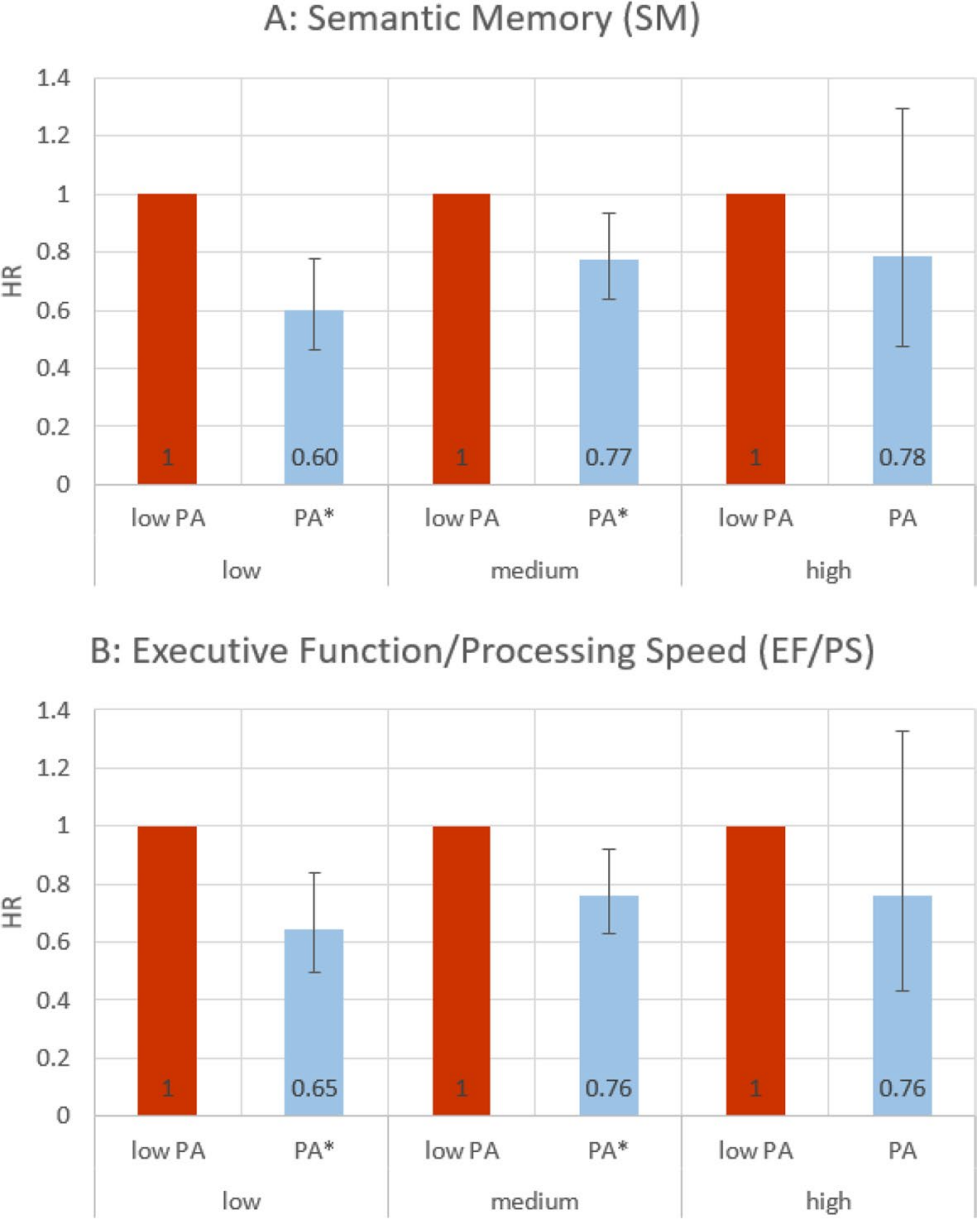
Results of Cox proportional hazard models with interaction term (combined HR and 95%CI) Note: The graphs show the HR and 95%CI for levels of PA across levels of cognitive function; PA = Physical Activity; HR = Hazard Ratio; asterisks (*) indicate significant differences between low PA and PA (*p* < 0.05); models adjusted for age, age.^2^, sex, education, income, Charlson Comorbidity Index score, lifestyle risk index, German language proficiency, and place of examination. Results for missing categories (“missing/incomplete information”) not shown. Source: NAKO baseline and mortality follow-up (*N* = 28,892; py = 216,427) (own calculations)

As illustrated in Fig. 2, the disparities in the mortality risk by PA were evident across the levels of SM (top panel, model 2a) and EF/PS (bottom panel, model 2b). The main effects and interaction effects utilized in the calculations are shown in Additional file 3.

Across both cognitive domains, PA generally exhibited lower mortality risks compared to low PA, most pronounced in the low cognitive function groups. For individuals with low SM or EF/PS, the HRs for PA were below 1 (low SM*PA: HR = 0.60 [0.46; 0.77]; low EF/PS*PA: HR = 0.65 [0.50; 0.84]), suggesting a potential association between PA and a lower risk of mortality in these subgroups. In medium cognitive function groups the differences based on PA levels were less pronounced, and in high cognitive function groups they were insignificant. These findings may indicate that the association between PA and mortality varies by cognitive function level, with potentially stronger associations observed among individuals with lower cognitive function.

### Sex-differences in the association between cognition, physical activity and mortality

In our general model, women exhibited a significantly lower mortality risk compared to men (HR = 0.50 [0.45; 0.56], Additional file 2). However, our sex-stratified Cox models indicated that this discrepancy remained largely unexplained by cognitive status and PA, either individually or in combination. Table 2 presents the primary results for both men and women. The complete model results are presented in Additional file 4.

**Table 2:** Results of sex-stratified Cox proportional hazard models with and without interaction term (HR, 95%CI)

		Men				Women			
Variables		M1—Distinct effects^a^							
		HR	p	lower 95% CI	upper 95% CI	HR	p	lower 95% CI	upper 95% CI
Semantic Memory (SM)	Low (ref.)	1				1			
	Medium	0.93	0.308	0.80	1.07	0.84	0.193	0.65	1.09
	High	0.93	0.588	0.70	1.22	0.68	0.033	0.48	0.97
	Missing/incomplete	1.17	0.235	0.90	1.52	1.29	0.329	0.77	2.17
Executive Function/Proc. Speed (EF/PS)	Low (ref.)	1				1			
	Medium	0.71	< 0.001	0.61	0.84	0.62	< 0.001	0.49	0.79
	High	0.70	0.011	0.54	0.92	0.59	0.006	0.40	0.86
	Missing/incomplete	0.83	0.159	0.64	1.08	0.47	0.007	0.27	0.81
Physical Activity (PA)	Active (PA)	0.72	< 0.001	0.61	0.86	0.66	0.002	0.50	0.86
	Low PA (ref.)	1				1			
	Missing/incomplete	0.84	0.131	0.67	1.05	0.85	0.306	0.61	1.17
		M2a: Interaction SM*PA							
Semantic Memory (SM) Main effects	Low (ref.)	1.00				1			
	Medium	0.80	0.172	0.58	1.10	0.57	0.035	0.34	0.96
	High	0.64	0.244	0.31	1.35	0.57	0.143	0.26	1.21
	Missing/incomplete	1.13	0.563	0.75	1.72	0.82	0.622	0.38	1.79
Physical Activity (PA) Main effects	Active (PA)	0.67	0.007	0.50	0.90	0.36	< 0.001	0.21	0.64
	Low PA (ref.)	1				1			
	Missing/incomplete	0.59	0.016	0.39	0.91	0.90	0.739	0.49	1.67
Semantic Memory*PA Interaction effects	Medium*Active	1.14	0.476	0.80	1.63	2.04	0.026	1.09	3.80
	Medium*Missing/Incomplete	1.59	0.088	0.93	2.71	0.86	0.709	0.40	1.85
	High*Active	1.43	0.373	0.65	1.63	1.73	0.215	0.73	4.12
	High*Missing/Incomplete	2.51	0.102	0.83	7.58	0.14	0.071	0.02	1.18
	Missing/Incomplete*Active	0.93	0.766	0.57	1.51	1.88	0.159	0.78	4.53
	Miss.*Miss.	1.57	0.175	0.82	3.01	1.58	0.352	0.60	4.16
		M2b: Interaction EF/PS*PA							
Executive Function/Proc. Speed (EF/PS) Main effects	Low (ref.)	1.00				1			
	Medium	0.56	< 0.001	0.41	0.77	0.70	0.159	0.43	1.15
	High	0.69	0.285	0.35	1.36	0.42	0.111	0.14	1.22
	Missing/incomplete	0.88	0.556	0.57	1.35	0.42	0.043	0.18	0.97
Physical Activity (PA) Main effects	Active (PA)	0.63	0.003	0.47	0.86	0.66	0.117	0.40	1.11
	Low PA (ref.)	1.00				1			
	Missing/incomplete	0.66	0.049	0.43	1.00	0.98	0.933	0.55	1.74
Executive Function/Proc. Speed*PA Interaction effects	Medium*Active	1.32	0.144	0.91	1.90	0.91	0.760	0.52	1.62
	Medium*Missing/Incomplete	1.48	0.142	0.88	2.50	0.64	0.240	0.31	1.34
	High*Active	1.04	0.915	0.50	2.15	1.48	0.506	0.47	4.65
	High*Missing/Incomplete	0.77	0.681	0.22	2.65	1.64	0.518	0.37	7.39
	Missing/Incomplete*Active	0.82	0.439	0.50	1.35	1.02	0.974	0.40	2.55
	Miss.*Miss.	1.47	0.253	0.76	2.82	1.48	0.456	0.53	4.20
Subjects		14,665				14,227			
Failures (Deaths)		1,097				508			
Observations (py)		108,914				107,513			
Time at risk		101,184				100,350			
LR chi^2^		648/654/656				310/328/315			

*PA* Physical Activity, *HR* Hazard Ratio, *95%CI* 95% Confidence Interval

^a^Effects of the main model without interaction terms; models adjusted for age, age^2^, sex, education, income, Charlson Comorbidity Index score, lifestyle risk factors, German language proficiency, and place of examination. The distinct effects and interaction effects were estimated in separate models

Source: NAKO baseline and mortality follow-up (men: *N* = 14,665; py = 108,914; women: *N* = 14,227; py = 107,513) (own calculations)

In the sex-stratified models (model 1, Table 2), elevated levels of EF/PS and PA were consistently associated with diminished mortality risk in both men and women. For instance, high EF/PS levels were associated with a HR of 0.70 [0.54; 0.92] in men and 0.59 [0.40; 0.86] in women. PA exerted a protective effect in both sexes (men: HR = 0.72 [0.61; 0.84]; women: HR = 0.66 [0.50; 0.86]). SM demonstrated a weaker and less consistent association, reaching significance only among women at the high level (HR = 0.68 [0.48; 0.97]).

In the interaction models (M2a and M2b, Table 2), combinations of high cognitive function and PA were generally associated with the lowest mortality risks, but the interactions of cognitive function and PA were largely insignificant, and the patterns were broadly similar for men and women. While the effect estimates for women were numerically lower in several instances, indicating stronger protective effects, the three-way interaction term revealed that there were not statistically significant sex-differences (p > 0.05).

### Sensitivity analyses

The results of our sensitivity analyses demonstrated the robustness of the primary findings. The models with a reduced set of covariates exhibited stronger associations between the cognitive domains, PA and mortality. In general, socio-economic characteristics partly explained the associations of the cognitive domains (models S1a/b), while health-related covariates partially explained the mortality disadvantage of those with low PA (models S2a/b). Subsequent to the adjustment of socio-economic covariates, the association between medium levels of SM and mortality became insignificant. The role of socio-economic characteristics was particularly pronounced among men, for whom no notable effect of SM was discernible following the adjustment for education and income. The analyses revealed that health-related characteristics accounted for approximately 50% of the observed mortality differences associated with PA, although the associations remained statistically significant. In women, the magnitude of the observed associations appeared to be less contingent on the specific modeling approach employed. Continuous measures of cognitive function and amount of PA (measured in MET) indicated significant associations (SM: HR = 0.96 [0.93; 0.99]; EF/PS: HR = 0.91 [0.88; 0.94]; PA-MET: HR = 1.00; models S3a/b). The results remained robust when individuals with a history of stroke (models S4a/b) or diabetes (models S5a/b) were excluded. The alteration of the censoring date (from December 31, 2021 onwards) led to a decline in the number of deaths, resulting in a lower statistical power but otherwise comparable outcomes. All results are demonstrated in Additional File 5.

## Discussion

This study examined the relationship between cognitive function, physical activity and all-cause mortality in the population of Germany aged 65 and older, with a particular focus on how physical activity modifies cognition-related mortality differences.

The findings indicated that low executive function/processing speed and low physical activity were associated with increased mortality by each around 30%. A protective effect was observed for high semantic memory, albeit to a lesser extent. Furthermore, the combination of low cognitive function (in both domains) and low activity yielded the highest mortality risk. However, the effect of physical activity was most pronounced among individuals with low cognition. In these groups, physical activity has been demonstrated to attenuate the risk of mortality by approximately 40% (semantic memory) and 35% (executive function/processing speed). The effect sizes did not differ significantly between men and women. The effect sizes did not differ significantly between men and women.

In men, semantic memory did not predict mortality. However, higher executive function/processing speed and physical activity were associated with reduced mortality. In women, high semantic memory, medium and high executive function/processing speed, and physical activity were associated with lower mortality. Mortality differences by cognitive function and physical activity were slightly more pronounced among women. The interaction analyses demonstrated that in men, physical activity was associated with a lower mortality risk at low and medium semantic memory levels, as well as low executive function/processing speed. In women, this protective effect was observed at low levels of semantic memory and medium levels of executive function/processing speed.

These findings underscore the significance of preserving cognitive function and maintaining physical activity in older adults to foster healthy aging and curtail (premature) mortality. They further suggest that physical activity may moderate the increased mortality risk observed in individuals with low cognitive function in both sexes.

Our study confirms the mortality disadvantages associated with pathological cognitive impairments, such as those observed in dementia [31, 34]. Consistent with prior research, our findings demonstrate that even individuals in the early or preliminary stages of cognitive impairment, below-average cognitive performance or cognitive decline [1, 46] face elevated mortality risks. Our analyses account for mild degrees of cognitive impairment and differentiate two cognitive domains. The negligible correlation between semantic memory and executive function/processing speed indicates that cognitive function is broad and that these domains assess different abilities. Consequently, they contribute differently to mortality differentials. Our domain-dependent approach underscores this and indicates that executive function/processing speed is more predictive for mortality than semantic memory [21, 27]. Specifically, our study confirms the strong association between executive function and mortality reported by others [60], which persists even after adjusting for physical activity and covariates.

Notably, in older individuals, objective cognition levels may not accurately reflect (perceived) limitations or decline [25, 26]. In line with Hayat et al. [27], this suggests that our measures may serve as proxies for future cognitive and health-related changes rather than as direct reflections of current impairments.

The mortality gradients for physical activity exhibited greater robustness compared to those observed for cognition. Low physical activity often accompanies sedentary behavior – prolonged lying, sitting, or reclining – although their direct correlation remains debated [45, 51]. Physical inactivity may also involve reduced social contacts, social control and environmental influences, and stimulation, all linked to higher mortality risks [3, 16, 17, 28]. Furthermore, social isolation and prolonged periods spent at home may worsen unmet health needs [7]. However, when interpreting the physical activity effect estimate, it is important to recognize that the 600 MET minutes/week threshold involves heterogeneous comparison groups. Comparing inactive individuals with highly active ones would likely reveal even larger mortality differences [48].

### Study strengths

Primary strengths of this study are the substantial sample size of the NAKO cohort and its linkage with the MoFU. This combination enabled the execution of differentiated analyses, including sex-stratified models, and facilitated the attainment of a longitudinal perspective that is seldom available in comparable population-based research.

Moreover, a significant strength of this study is the utilization of validated measures for physical activity via GPAQ [72], which is accompanied by a standardized recording and threshold of physical activity. This methodological approach ensures transferability, transparency, and comparability. However, to attain more detailed results, subsequent studies could differentiate between the activity subdomains [69]. Alternative metrics like Life’s Essential 8 (LE8) could provide a broader perspective on lifestyle dimensions and prevention [43]. However, we focused on physical activity as the primary exposure, while carefully accounting for other health- and lifestyle-related factors, allowing a more precise assessment of its independent association with all-cause mortality.

The assessment of cognitive function across two distinct domains has been shown to be more efficient than the evaluation of a single global indicator. This approach facilitated the demonstration of domain-specific associations, both in terms of mortality differentials and the underlying mechanisms. In accordance with the findings of preceding studies [27, 29, 57, 58], the present study demonstrates a robust correlation between executive function/processing speed and mortality.

Another strength of this study is the broad set of covariates, which serve to clarify mortality differentials and the associations of physical activity and cognitive function. However, certain potential factors, such as comprehensive CCI information, living environment, biographical details, and subjective health indicators, were not included. Future studies should integrate these dimensions to enhance robustness.

Finally, the incorporation of sex stratification facilitates a more profound comprehension of sex-specific and global associations.

### Study limitations

A limitation of this study is the cross-sectional design of the NAKO baseline examination, which prevents temporal differentiation between cognitive function and physical activity and precludes formal mediation analyses. Prior research suggests reciprocal associations [9, 19], with findings indicating physical activity can predict cognition [3, 17], and vice versa [19]. While this study provides important insights into the associations between cognitive function, physical activity, and mortality, subsequent longitudinal analyses are needed to establish causal pathways and formally examine potential mediation. However, by linking baseline and the NAKO MoFu, we could identify mortality trajectories prospectively. Consequently, these trajectories can be interpreted as subsequent in time and hence as potential consequences of physical activity and cognition, although they may also reflect proximity to death, and hence indicate reverse causality [30, 47, 71]. It is also plausible that third factors were in play, exerting influence on physical activity, cognition, and mortality. Despite our efforts to adjust for potential confounders, the possibility of residual confounding remains a concern.

Regarding mortality, only few deaths were observed soon after the baseline, and our cohort mortality rate was lower than in the population in Germany, potentially reflecting a "healthy volunteer bias" [4]. Timing of death cases may also reflect a reporting lag,right-censoring was applied from December 31, 2023. A more rigorous censoring approach, as implemented in our sensitivity analysis censoring from December 31, 2021, yielded comparable results and does not indicate selective bias. However, future NAKO data will enhance mortality ascertainment and bias evaluation.

Furthermore, the sample’s health profile suggests a healthy volunteer bias [4] or a social desirability bias [37], which may also have affected the measurement of physical activity via GPAQ [5, 39]. Time‐lagged effects of cognition, physical activity, and covariates on mortality cannot yet be assessed given the age range (65–75 years at baseline) and limited follow‐up, potentially underestimating long‐term associations.

Finally, a critical aspect of this study lies in the handling of missing data for the primary exposures of physical activity and cognition. We cautiously treated missing values as a separate category. This expanded approach of available case analysis appears appropriate, as the nature of missingness cannot be assessed and the affected variables are included as either outcomes or exposures in the regression models [36]. To address potential residual bias, we adjusted for relevant characteristics in our models.

## Conclusions

This study provides novel insights into the sex-specific effects of physical activity and cognitive function on mortality among older adults in Germany. Individuals exhibiting low physical activity and low levels of executive function and processing speed have elevated mortality risks. The tendency of lower mortality risks in individuals with higher levels of semantic memory observed in the whole sample is only evident in the female subsample. However, no significant sex differences were observed with regard to the interaction of cognitive function and physical activity. The associations are not explained by socio-economic characteristics and health characteristics. The findings indicate a potential compensatory effect of physical activity in both sexes, as weaker and largely insignificant mortality differences were observed in the physically active groups. Future research and longitudinal analyses are necessary to elucidate the underlying pathways.

## Supplementary Information

Additional file 1. Descriptive statistics and bivariate associations for the analytic sample, men, and women.

Additional file 2. Results of Cox proportional hazard model for mortality – full model.

Additional file 3. Results of Cox proportional hazard models with interaction term (main effects and interaction effects; HR, 95%CI).

Additional file 4. Results of Cox proportional hazard models for mortality with distinct effects for men and women.

Additional file 5. Sensitivity Analyses - Results of Cox proportional hazard models with interaction term (main effects and interaction effects; HR, 95%CI).

## Data Availability

The datasets analyzed during our study are not publicly available due to privacy concerns and strict data protection regulations. Data are available upon reasonable request via https://transfer.nako.de.

## References

[1] Bae JB, Han JW, Kwak KP, Kim BJ, Kim SG, Kim JL, Kim TH, Ryu SH, Moon SW, Park JH [Joon Hyuk], Youn JC, Lee DY, Lee DW, Lee SB, Lee JJ, Jhoo JH, Kim KW. Impact of Mild Cognitive Impairment on Mortality and Cause of Death in the Elderly. Journal of Alzheimer's Disease : JAD. 2018;64(2), 607–616. 10.3233/JAD-171182 10.3233/JAD-171182 29914024

[2] Batty GD, Deary IJ, Zaninotto P. Association of cognitive function with cause-specific mortality in middle and older age: follow-up of participants in the English Longitudinal Study of Ageing. Am J Epidemiol. 2016;183(3):183–90. 10.1093/aje/kwv139. 26803665 10.1093/aje/kwv139 PMC4724091

[3] Blondell SJ, Hammersley-Mather R, Veerman JL. Does physical activity prevent cognitive decline and dementia? A systematic review and meta-analysis of longitudinal studies. BMC Public Health. 2014;14:510. 10.1186/1471-2458-14-510. 24885250 10.1186/1471-2458-14-510 PMC4064273

[4] Brayne C, Moffitt TE. The limitations of large-scale volunteer databases to address inequalities and global challenges in health and aging. Nat Aging. 2022;2(9):775–83. 10.1038/s43587-022-00277-x. 37118500 10.1038/s43587-022-00277-x PMC10154032

[5] Brenner PS, DeLamater JD. Social desirability bias in self-reports of physical activity: is an exercise identity the culprit? Soc Indic Res. 2014;117(2):489–504. 10.1007/s11205-013-0359-y.

[6] Burton R, Fryers PT, Sharpe C, Clarke Z, Henn C, Hydes T, et al. The independent and joint risks of alcohol consumption, smoking, and excess weight on morbidity and mortality: a systematic review and meta-analysis exploring synergistic associations. Public Health. 2024;226:39–52. 10.1016/j.puhe.2023.10.035. 38000113 10.1016/j.puhe.2023.10.035

[7] Chamberlain S, Savage RD, Bronskill SE, Griffith LE, Rochon P, Batara J, et al. Retrospective cross-sectional study examining the association between loneliness and unmet healthcare needs among middle-aged and older adults using the Canadian Longitudinal Study of Aging (CLSA). BMJ Open. 2023;13(3):e068769. 10.1136/bmjopen-2022-068769. 36918248 10.1136/bmjopen-2022-068769 PMC10016309

[8] Charlson ME, Carrozzino D, Guidi J, Patierno C. Charlson comorbidity index: a critical review of clinimetric properties. Psychother Psychosom. 2022;91(1):8–35. 10.1159/000521288. 34991091 10.1159/000521288

[9] Choudhary, A. K., Loganathan, S., & Maheshkumar, K. (2021). A Sedentary Lifestyle and Cognitive Function. In D. W. M. Md Saad (Ed.), Challenges in Disease and Health Research Vol. 6 (pp. 52–62). Book Publisher International (a part of SCIENCEDOMAIN International). 10.9734/bpi/cdhr/v6/7457D

[10] Compernolle S, Bourdeaudhuij I, Cardon G, van Dyck D. Sex-specific typologies of older adults’ sedentary behaviors and their associations with health-related and socio-demographic factors: a latent profile analysis. BMC Geriatr. 2021;21(1):66. 10.1186/s12877-021-02011-5. 33468055 10.1186/s12877-021-02011-5 PMC7816402

[11] Cooper C, Sommerlad A, Lyketsos CG, Livingston G. Modifiable predictors of dementia in mild cognitive impairment: a systematic review and meta-analysis. Am J Psychiatry. 2015;172(4):323–34. 10.1176/appi.ajp.2014.14070878. 25698435 10.1176/appi.ajp.2014.14070878

[12] Davis D, Cooper R, Terrera GM, Hardy R, Richards M, Kuh D. Verbal memory and search speed in early midlife are associated with mortality over 25 years’ follow-up, independently of health status and early life factors: a British birth cohort study. Int J Epidemiol. 2016;45(4):1216–25. 10.1093/ije/dyw100. 27498153 10.1093/ije/dyw100 PMC6639118

[13] Davis MG, Fox KR, Hillsdon M, Sharp DJ, Coulson JC, Thompson JL. Objectively measured physical activity in a diverse sample of older urban UK adults. Med Sci Sports Exerc. 2011;43(4):647–54. 10.1249/MSS.0b013e3181f36196. 20689449 10.1249/MSS.0b013e3181f36196

[14] Ding D, Nguyen B, Nau T, Luo M, Del Pozo Cruz B, Dempsey PC, et al. Daily steps and health outcomes in adults: a systematic review and dose-response meta-analysis. Lancet Public Health. 2025;10(8):e668–81. 10.1016/S2468-2667(25)00164-1. 40713949 10.1016/S2468-2667(25)00164-1

[15] Ekelund U, Tarp J, Steene-Johannessen J, Hansen BH, Jefferis B, Fagerland MW, et al. Dose-response associations between accelerometry measured physical activity and sedentary time and all cause mortality: systematic review and harmonised meta-analysis. BMJ. 2019;366:l4570. 10.1136/bmj.l4570. 31434697 10.1136/bmj.l4570 PMC6699591

[16] Ellwardt L, van Tilburg T, Aartsen M, Wittek R, Steverink N. Personal networks and mortality risk in older adults: a twenty-year longitudinal study. PLoS ONE. 2015;10(3):e0116731. 10.1371/journal.pone.0116731. 25734570 10.1371/journal.pone.0116731 PMC4348168

[17] Erickson KI, Hillman C, Stillman CM, Ballard RM, Bloodgood B, Conroy DE, et al. Physical activity, cognition, and brain outcomes: a review of the 2018 physical activity guidelines. Med Sci Sports Exerc. 2019;51(6):1242–51. 10.1249/MSS.0000000000001936. 31095081 10.1249/MSS.0000000000001936 PMC6527141

[18] Esteban-Cornejo I, Cabanas-Sánchez V, Higueras-Fresnillo S, Ortega FB, Kramer AF, Rodriguez-Artalejo F, et al. Cognitive frailty and mortality in a national cohort of older adults: the role of physical activity. Mayo Clin Proc. 2019;94(7):1180–9. 10.1016/j.mayocp.2018.10.027. 30871783 10.1016/j.mayocp.2018.10.027

[19] Falck RS, Landry GJ, Best JR, Davis JC, Chiu BK, Liu-Ambrose T. Cross-sectional relationships of physical activity and sedentary behavior with cognitive function in older adults with probable mild cognitive impairment. Phys Ther. 2017;97(10):975–84. 10.1093/ptj/pzx074. 29029554 10.1093/ptj/pzx074 PMC5803762

[20] Fried LP, Kronmal RA, Newman AB, Bild DE, Mittelmark MB, Polak JF, et al. Risk factors for 5-year mortality in older adults: the Cardiovascular Health Study. JAMA. 1998;279(8):585–92. 10.1001/jama.279.8.585. 9486752 10.1001/jama.279.8.585

[21] Gathright EC, Dolansky MA, Gunstad J, Josephson RA, Moore SM, Hughes JW. Examination of attention, executive function, and memory as predictors of mortality risk in adults with systolic heart failure. Eur J Cardiovasc Nurs. 2019;18(8):729–35. 10.1177/1474515119863182. 31342781 10.1177/1474515119863182 PMC6916714

[22] Ghisletta P, Aichele S, Gerstorf D, Carollo A, Lindenberger U. Verbal fluency selectively predicts survival in old and very old age. Psychol Sci. 2025;36(2):87–101. 10.1177/09567976241311923. 39992974 10.1177/09567976241311923 PMC13428923

[23] Goldman N, Glei DA, Weinstein M. The Best Predictors of Survival: Do They Vary by Age, Sex, and Race? Popul Dev Rev. 2017;43(3):541–60. 10.1111/padr.12079. 29398738 10.1111/padr.12079 PMC5791760

[24] Guthold R, Stevens GA, Riley LM, Bull FC. Worldwide trends in insufficient physical activity from 2001 to 2016: a pooled analysis of 358 population-based surveys with 1·9 million participants. Lancet Glob Health. 2018;6(10):e1077–86. 10.1016/S2214-109X(18)30357-7. 30193830 10.1016/S2214-109X(18)30357-7

[25] Hallowell ES, van Patten R. Clinical utility of self-reported and informant-reported cognitive complaints in older adults. Int Psychogeriatr. 2022;34(12):1007–10. 10.1017/S1041610222000643. 35882428 10.1017/S1041610222000643

[26] Harris S, Gehling J, Marx H, Dhaliwal R, Robertson F, Chenoweth CJ. Bridging perspective: a comparative examination of subjective memory and ageing measures. Innov Aging. 2024;8(1):8–9. 10.1093/geroni/igae098.0025.

[27] Hayat SA, Luben R, Dalzell N, Moore S, Hogervorst E, Matthews FE, et al. Understanding the relationship between cognition and death: a within cohort examination of cognitive measures and mortality. Eur J Epidemiol. 2018;33(11):1049–62. 10.1007/s10654-018-0439-z. 30203336 10.1007/s10654-018-0439-z PMC6208995

[28] Holt-Lunstad J, Smith TB, Baker M, Harris T, Stephenson D. Loneliness and social isolation as risk factors for mortality: a meta-analytic review. Perspect Psycholog Sci. 2015;10(2):227–37. 10.1177/1745691614568352. 10.1177/1745691614568352 25910392

[29] Johnson JK, Lui LY, Yaffe K. Executive function, more than global cognition, predicts functional decline and mortality in elderly women. The Journals of Gerontology. Series A Biol Sci Med Sci. 2007;62(10):1134–41. 10.1093/gerona/62.10.1134. 10.1093/gerona/62.10.1134 PMC2049089 17921427

[30] Karunananthan S, Moodie EEM, Bergman H, Payette H, Wolfson D, Diehr PH, et al. The association between physical function and proximity to death in older adults: a multilevel analysis of 4,150 decedents from the Cardiovascular Health Study. Ann Epidemiol. 2019;35:59-65.e5. 10.1016/j.annepidem.2019.04.005. 31221508 10.1016/j.annepidem.2019.04.005

[31] Kim D. Correlation between physical function, cognitive function, and health-related quality of life in elderly persons. J Phys Ther Sci. 2016;28(6):1844–8. 10.1589/jpts.28.1844. 27390430 10.1589/jpts.28.1844 PMC4932071

[32] Kleineidam L, Stark M, Riedel-Heller SG, Pabst A, Schmiedek F, Streit F, et al. The assessment of cognitive function in the German National Cohort (NAKO) - associations of demographics and psychiatric symptoms with cognitive test performance. World J Biol Psychiatry. 2023;24(10):909–23. 10.1080/15622975.2021.2011408. 35175181 10.1080/15622975.2021.2011408

[33] Kohl HW, Craig CL, Lambert EV, Inoue S, Alkandari JR, Leetongin G, et al. The pandemic of physical inactivity: global action for public health. Lancet. 2012;380(9838):294–305. 10.1016/S0140-6736(12)60898-8. 22818941 10.1016/S0140-6736(12)60898-8

[34] Kramarow, E., & Betzaida, T.‑V. (2024). Dementia Mortality Among Adults Age 65 and Older: United States, 2018–2022. 10.15620/cdc/165795

[35] Kraus WE, Powell KE, Haskell WL, Janz KF, Campbell WW, Jakicic JM, et al. Physical activity, all-cause and cardiovascular mortality, and cardiovascular disease. Med Sci Sports Exerc. 2019;51(6):1270–81. 10.1249/MSS.0000000000001939. 31095084 10.1249/MSS.0000000000001939 PMC6527136

[36] Kuss O, Becher H, Wienke A, Ittermann T, Ostrzinski S, Schipf S, et al. Statistical analysis in the German National Cohort (NAKO) - specific aspects and general recommendations. Eur J Epidemiol. 2022;37(4):429–36. 10.1007/s10654-022-00880-7. 35653006 10.1007/s10654-022-00880-7 PMC9187540

[37] Latkin CA, Edwards C, Davey-Rothwell MA, Tobin KE. The relationship between social desirability bias and self-reports of health, substance use, and social network factors among urban substance users in Baltimore, Maryland. Addict Behav. 2017;73:133–6. 10.1016/j.addbeh.2017.05.005. 28511097 10.1016/j.addbeh.2017.05.005 PMC5519338

[38] Lee I‑M, Shiroma EJ, Lobelo F, Puska P, Blair SN, Katzmarzyk PT. Effect of physical inactivity on major non-communicable diseases worldwide: an analysis of burden of disease and life expectancy. Lancet. 2012;380(9838):219–29. 10.1016/S0140-6736(12)61031-9. 22818936 10.1016/S0140-6736(12)61031-9 PMC3645500

[39] Leitzmann M, Gastell S, Hillreiner A, Herbolsheimer F, Baumeister SE, Bohn B, et al. Körperliche Aktivität in der NAKO Gesundheitsstudie: erste Ergebnisse des multimodalen Erhebungskonzepts [Physical activity in the German National Cohort (NAKO): use of multiple assessment tools and initial results]. Bundesgesundheitsblatt Gesundheitsforschung Gesundheitsschutz. 2020;63(3):301–11. 10.1007/s00103-020-03099-7. 32055903 10.1007/s00103-020-03099-7

[40] Levine DA, Gross AL, Briceño EM, Tilton N, Giordani BJ, Sussman JB, et al. Sex differences in cognitive decline among US adults. JAMA Netw Open. 2021;4(2):e210169. 10.1001/jamanetworkopen.2021.0169. 33630089 10.1001/jamanetworkopen.2021.0169 PMC7907956

[41] Li C‑L, Chiu Y‑C, Shyu Y‑IL, Stanaway FF, Chang H‑Y, Bai Y‑B. Does physical activity protect older persons with frailty and cognitive impairment from excess all-cause mortality? Arch Gerontol Geriatr. 2021;97:104500. 10.1016/j.archger.2021.104500. 34388680 10.1016/j.archger.2021.104500

[42] Li, H., Zheng, Y., Li, Q., & Wang, M. (2024). Cognitive Function, Healthy Lifestyle, and All-Cause Mortality among Chinese Older Adults: A Longitudinal Prospective Study. Nutrients, 16(9). 10.3390/nu16091297 10.3390/nu16091297 PMC11085585 38732544

[43] Lloyd-Jones DM, Allen NB, Anderson CAM, Black T, Brewer LC, Foraker RE, et al. Life’s essential 8: updating and enhancing the American Heart Association’s construct of cardiovascular health: a presidential advisory from the American Heart Association. Circulation. 2022;146(5):e18–43. 10.1161/CIR.0000000000001078. 35766027 10.1161/CIR.0000000000001078 PMC10503546

[44] Loef M, Walach H. The combined effects of healthy lifestyle behaviors on all cause mortality: a systematic review and meta-analysis. Prev Med. 2012;55(3):163–70. 10.1016/j.ypmed.2012.06.017. 22735042 10.1016/j.ypmed.2012.06.017

[45] Lord S, Chastin SFM, McInnes L, Little L, Briggs P, Rochester L. Exploring patterns of daily physical and sedentary behaviour in community-dwelling older adults. Age Ageing. 2011;40(2):205–10. 10.1093/ageing/afq166. 21239410 10.1093/ageing/afq166

[46] Lv X, Li W, Ma Y, Chen H, Zeng Y, Yu X, et al. Cognitive decline and mortality among community-dwelling Chinese older people. BMC Med. 2019;17(1):63. 10.1186/s12916-019-1295-8. 30871536 10.1186/s12916-019-1295-8 PMC6419492

[47] MacDonald SWS, Hultsch DF, Dixon RA. Aging and the shape of cognitive change before death: terminal decline or terminal drop? J Gerontol B Psychol Sci Soc Sci. 2011;66(3):292–301. 10.1093/geronb/gbr001. 21300703 10.1093/geronb/gbr001 PMC3078759

[48] Martinez-Gomez D, Luo M, Huang Y, Rodríguez-Artalejo F, Ekelund U, Sotos-Prieto M, et al. Physical activity and all-cause mortality by age in 4 multinational megacohorts. JAMA Netw Open. 2024;7(11):e2446802. 10.1001/jamanetworkopen.2024.46802. 39570587 10.1001/jamanetworkopen.2024.46802 PMC11582934

[49] Murayama, H., Sugiyama, M., Inagaki, H., Ura, C., Miyamae, F., Edahiro, A., Motokawa, K., Okamura, T., & Awata, S. (2023). The Relationship Between Cognitive Decline and All-Cause Mortality Is Modified by Living Alone and a Small Social Network: A Paradox of Isolation. The Journals of Gerontology. Series B, Psychological Sciences and Social Sciences, 78(11), 1927–1934. 10.1093/geronb/gbad134 10.1093/geronb/gbad134 37725961

[50] Oksuzyan, A., Juel, K., Vaupel, J. W., & Christensen, K. (2008). Men: Good health and high mortality. Sex differences in health and aging. Aging Clinical and Experimental Research, 20(2), 91–102. 10.1007/BF03324754 10.1007/bf03324754 PMC3629373 18431075

[51] Panahi S, Tremblay A. Sedentariness and health: is sedentary behavior more than just physical inactivity? Front Public Health. 2018;6:258. 10.3389/fpubh.2018.00258. 30250838 10.3389/fpubh.2018.00258 PMC6139309

[52] Park, J. H [Jung Ha], Moon, J. H., Kim, H. J., Kong, M. H., & Oh, Y. H. (2020). Sedentary Lifestyle: Overview of Updated Evidence of Potential Health Risks. Korean Journal of Family Medicine, 41(6), 365–373. 10.4082/kjfm.20.0165 10.4082/kjfm.20.0165 PMC7700832 33242381

[53] Peters A, Greiser KH, Göttlicher S, Ahrens W, Albrecht M, Bamberg F, et al. Framework and baseline examination of the German National Cohort (NAKO). Eur J Epidemiol. 2022;37(10):1107–24. 10.1007/s10654-022-00890-5. 36260190 10.1007/s10654-022-00890-5 PMC9581448

[54] Pinto AJ, Bergouignan A, Dempsey PC, Roschel H, Owen N, Gualano B, et al. Physiology of sedentary behavior. Physiol Rev. 2023;103(4):2561–622. 10.1152/physrev.00022.2022. 37326297 10.1152/physrev.00022.2022 PMC10625842

[55] Plassman BL, Williams JW, Burke JR, Holsinger T, Benjamin S. Systematic review: factors associated with risk for and possible prevention of cognitive decline in later life. Ann Intern Med. 2010;153(3):182–93. 10.7326/0003-4819-153-3-201008030-00258. 20547887 10.7326/0003-4819-153-3-201008030-00258

[56] Rau R, Schmertmann CP. District-level life expectancy in Germany. Dtsch Arztebl Int. 2020;117(29–30):493–9. 10.3238/arztebl.2020.0493. 33087229 10.3238/arztebl.2020.0493 PMC7588608

[57] Rostamian S, Haan S, van der Grond J, van Buchem MA, Ford I, Jukema JW, et al. Cognitive function in dementia-free subjects and survival in old age: the PROSPER study. Am J Med. 2019;132(12):1466-1474.e4. 10.1016/j.amjmed.2019.06.001. 31228412 10.1016/j.amjmed.2019.06.001

[58] Rostamian S, Le Cessie S, Marijt KA, Jukema JW, Mooijaart SP, van Buchem MA, et al. Association of cognitive function with increased risk of cancer death and all-cause mortality: longitudinal analysis, systematic review, and meta-analysis of prospective observational studies. PLoS ONE. 2022;17(1):e0261826. 10.1371/journal.pone.0261826. 34995287 10.1371/journal.pone.0261826 PMC8741047

[59] Rothman, K. J., Greenland, S., & Lash, T. L. (2008). Modern Epidemiology (3rd Edition). Lippincott, Williams & Wilkins.

[60] Shipley BA, Der G, Taylor MD, Deary IJ. Cognition and mortality from the major causes of death: the health and lifestyle survey. J Psychosom Res. 2008;65(2):143–52. 10.1016/j.jpsychores.2008.02.017. 18655859 10.1016/j.jpsychores.2008.02.017

[61] Simpson EEA, O’Connor JM, Livingstone MBE, Rae G, Stewart-Knox BJ, Andriollo-Sanchez M, et al. Health and lifestyle characteristics of older European adults: the ZENITH study. Eur J Clin Nutr. 2005;59(2):S13-21. 10.1038/sj.ejcn.1602292. 16254575 10.1038/sj.ejcn.1602292

[62] Strain T, Flaxman S, Guthold R, Semenova E, Cowan M, Riley LM, et al. National, regional, and global trends in insufficient physical activity among adults from 2000 to 2022: a pooled analysis of 507 population-based surveys with 5·7 million participants. Lancet Glob Health. 2024;12(8):e1232–43. 10.1016/S2214-109X(24)00150-5. 38942042 10.1016/S2214-109X(24)00150-5 PMC11254784

[63] Tamosiunas, A., Sapranaviciute-Zabazlajeva, L., Luksiene, D., Virviciute, D., & Bobak, M. (2020). Cognitive Function and Mortality: Results from Kaunas HAPIEE Study 2006-2017. Int J Environ Res Public Health, 17(7). 10.3390/ijerph17072397 10.3390/ijerph17072397 PMC7178058 32244660

[64] Twersky, S. E., Jefferson, R., Garcia-Ortiz, L., Williams, E., & Pina, C. (2024). The Impact of Limited English Proficiency on Healthcare Access and Outcomes in the U.S.: A Scoping Review. Healthcare (Basel, Switzerland), 12(3). 10.3390/healthcare12030364 10.3390/healthcare12030364 PMC10855368 38338249

[65] VanderWeele TJ, Shpitser I. A new criterion for confounder selection. Biometrics. 2011;67(4):1406–13. 10.1111/j.1541-0420.2011.01619.x. 21627630 10.1111/j.1541-0420.2011.01619.x PMC3166439

[66] Vargas-Torres-Young DA, Salazar-Talla L, Cuba-Ruiz S, Urrunaga-Pastor D, Runzer-Colmenares FM, Parodi JF. Cognitive frailty as a predictor of mortality in older adults: a longitudinal study in Peru. Front Med. 2022;9:910005. 10.3389/fmed.2022.910005. 10.3389/fmed.2022.910005 PMC9256954 35814770

[67] Wang K, Fang Y, Zheng R, Zhao X, Wang S, Lu J, et al. Associations of socioeconomic status and healthy lifestyle with incident dementia and cognitive decline: two prospective cohort studies. EClinMed. 2024;76:102831. 10.1016/j.eclinm.2024.102831. 10.1016/j.eclinm.2024.102831 PMC11420443 39318786

[68] Wang, T., Li, Y., & Zheng, X. (2023). Association of socioeconomic status with cardiovascular disease and cardiovascular risk factors: A systematic review and meta-analysis. Zeitschrift Fur Gesundheitswissenschaften = Journal of Public Health, 1–15. 10.1007/s10389-023-01825-4 10.1007/s10389-023-01825-4 PMC9867543 36714072

[69] Wanner M, Tarnutzer S, Martin BW, Braun J, Rohrmann S, Bopp M, et al. Impact of different domains of physical activity on cause-specific mortality: a longitudinal study. Prev Med. 2014;62:89–95. 10.1016/j.ypmed.2014.01.025. 24513168 10.1016/j.ypmed.2014.01.025

[70] Whitley E, Deary IJ, Ritchie SJ, Batty GD, Kumari M, Benzeval M. Variations in cognitive abilities across the life course: cross-sectional evidence from Understanding Society: the UK household longitudinal study. Intelligence. 2016;59:39–50. 10.1016/j.intell.2016.07.001. 27932853 10.1016/j.intell.2016.07.001 PMC5127898

[71] Wilson RS, Beckett LA, Bienias JL, Evans DA, Bennett DA. Terminal decline in cognitive function. Neurology. 2003;60(11):1782–7. 10.1212/01.wnl.0000068019.60901.c1. 12796531 10.1212/01.wnl.0000068019.60901.c1

[72] World Health Organization.. Global Physical Activity Questionnaire (GPAQ): Analysis Guide. 2010. https://www.who.int/docs/default-source/ncds/ncd-surveillance/gpaq-analysis-guide.pdf.

[73] Yaffe K, Peltz CB, Ewing SK, McCulloch CE, Cummings SR, Cauley JA, et al. Long-term cognitive trajectories and mortality in older women. J Gerontol A Biol Sci Med Sci. 2016;71(8):1074–80. 10.1093/gerona/glw003. 26843186 10.1093/gerona/glw003 PMC4945886

[74] Zhang XM, Wu XJ, Cao J, Jiao J, Chen W. Association between cognitive frailty and adverse outcomes among older adults: a meta-analysis. J Nutr Health Aging. 2022;26(9):817–25. 10.1007/s12603-022-1833-5. 36156673 10.1007/s12603-022-1833-5 PMC12280716

